# Ultrastructural and morphometrical changes of mice ovaries following experimentally induced copper poisoning

**Published:** 2012-09-30

**Authors:** H Babaei, L Roshangar, E Sakhaee, J Abshenas, R Kheirandish, R Dehghani

**Affiliations:** 1 Department of Clinical Sciences, Faculty of Veterinary Medicine, Shahid Bahonar University of Kerman, Kerman, IRAN; 2 Neuroscience Research Center, Tabriz University of Medical Sciences, Tabriz, IRAN; 3 Department of Pathobiology, Faculty of Veterinary Medicine, Shahid Bahonar University of Kerman, Kerman, IRAN.; 4 Graduated Student of Faculty of Veterinary Medicine, Shahid Bahonar University of Kerman, Kerman, IRAN

**Keywords:** Copper poisoning, Electron microscopy, Morphometry, Ovary, Mice

## Abstract

**Background:**

Copper (Cu) is an essential trace element involved in normal reproduction but its overexposure may produce some detrimental effects. The aim of this study was to investigate the effects of copper sulfate poisoning on morphometery of mice ovarian structures and probable intracellular changes.

**Methods:**

Thirty mature female mice were randomly allocated to control and two treatment groups. In treatment groups, two different doses of copper sulfate including 100 mg/kg and 200 mg/kg in 0.2 cc were applied once a day for 35 consecutive days by gavage. Control animals received normal saline using the same volume and similar method. Animals from each experimental group were sacrificed 14 and 35 days after the beginning of drug administration and the left ovaries were removed for stereological evaluations by light microscopy and right ovaries were obtained for preparing electron microscopic sections.

**Results:**

The morphometrical results showed that only the number of antral follicles was decreased by 100 mg/kg copper sulfate on day 14 compared to the control group (P=0.043). Hence, higher copper dose or longer consumption period significantly reduced different classes of follicles and corpora lutea. With 100 mg/kg copper sulfate some mild ultrastructural cell damages such as decrease of zona pellucida thickness, limited vacuolated areas and nuclear envelop dilation were seen on day 14. Higher or longer Cu administration produced more detrimental effects including more vacuolated areas, presence of secondary lysosomes, irregularity in cell shape and segmented nuclei with condensed and marginated chromatin and more enlarged and damaged mitochondria.

**Conclusion:**

New evidences of early as well as late intracellular damages of copper has been presented by accurate stereological and ultrastructural methods. Antral follicles was the most susceptible cells with the lower and shorter copper consumption and long term or higher dose of copper affected the whole of ovarian structures.

## Introduction

Copper is an essential trace element involved in normal reproduction of mammals. (1) Despite of its necessity for different metabolic processes and enzyme activities, (2) Copper over-exposure may produce some detrimental effects for body. Usually, occupational exposure to Cu may lead to copper toxicosis in the industrial workers. (3) In animals, long-term intake of Cu compounds of different origin is the most common form of copper poisoning. It means that the animals are reared closed to industrial plants, and ingest Cu from industrial deposits through feed or from air throughout their entire life. (4) Copper regulation is controlled mainly by the liver, where it can be mobilized into the circulation or excreted via the bile. (5) In chronic copper poisoning, Cu is gradually deposited in the liver without producing any significant sign. When the hepatic copper storage capacity is exceeded, it may result in hepatocellular necrosis and consequently the liberation of Cu from the liver into the blood stream produces hemolysis, jaundice, and renal insufficiency. (1)

Clinical manifestations associated with Cu poisoning and its pathological features specially in organs such as liver, kidney, spleen, lung and intestine have been well demonstrated in animals. (4,6-8) Recently, the effect of copper poisoning on sperm quality and testicular histopathology has been investigated, (9) but unfortunately there is few information about the effects of Cu toxicosis on female genital organs. We know from early studies that Cu administration can induce ovulation in rabbits, (10,11) and ewes, (12) or Cu can stimulate both basal and gonadotrophin releasing hormone (GnRH)-stimulated LH release from pituitary cells of immature female rats. (13)

According to our literature reviews, we could not interestingly find any experimental information about ultrastructural and morphometrical changes of ovaries during Cu poisoning. So, in the present study, the effects of copper sulfate administration on mice ovaries was investigated by providing electron microscopic and histopathological sections.

## Materials and Methods

### Animals

NMRI mature (6 weeks old) female mice (30-35 gr) were purchased from Razi Research Institute of Kerman, Iran and kept in the Center of Laboratory Animal Care at the Veterinary Faculty of Shahid Bahonar University of Kerman, Iran for 1 week before treatment. The mice were housed in groups of five per cage and maintained under standard laboratory conditions (12 h light: 12 h dark and 22±2 ºC) during the experimental period. During the study, the animals received water and pellet food (Javaneh Khorasan Co, Iran) ad libitum. All investigations were conducted in accordance with the Guiding Principles for the Care and use of Research Animals and were approved by the Animal Ethics Committee at the Veterinary Faculty of Shahid Bahonar University of Kerman, Iran.

### Study design

Thirty mature (6-8 weeks old) female mice were randomly allocated to either control (Con, n=6) or two treatment groups each containing twelve animals. To monitor the short and long-term effects of Cu on ovarian structure, two different doses of copper sulfate were applied once a day for 14 and 35 consecutive days by gavage. The first treatment group (Cu100, n=12) received copper sulfate at a dose of 100 mg/kg in 0.2 cc and the second treatment group (Cu200, n=12) was given copper sulfate at a dose of 200 mg/kg in 0.2 cc. Control animals received normal saline using the same volume and similar method. The dose of copper sulfate used in our experiment was according to the previous study for producing of copper poisoning in rats. (9) Animals from each experimental group were sacrificed upon diethyl ether anesthesia (May & Baker Ltd., Dagenham, England) by cervical dislocation 14 (n=6) and 35 (n=6) days after the beginning of copper sulfate administration, respectively.

### Sampling

Left ovaries were removed for histopathological evaluations and right ovaries were obtained from the mice in each group for preparing electron microscopic sections. Blood samples were obtained from each animal by cardiac puncture on days 14 and 35 following copper sulfate administration. Whole blood was collected aseptically using sterile 2 ml syringe and poured into tubes without anticoagulant. The blood was centrifuged (Universal 320R, Hettich, Germany) at 3000 g for 10 minutes at room temperature, and sera were harvested using disposable pipettes and transferred into 1.5 ml sterile micro tubes (Eppendorf). Serum samples were kept at −20ºC until the day of analysis. Serum Cu was analyzed by an atomic absorption spectrometer (Buck Scientific Co., USA).

### Morphometrical study

Preparation of ovarian slide samples were made according to our previous report. (14) Briefly, the left ovary of each group were fixed in Bouin’s solution, embedded in paraffin wax, sectioned serially at 5 µm thickness on a rotary microtome (Slee, Technik GMBH, Mainz, Germany) and stained with haematoxylin and eosin (H&E, Merck, Germany). Differential structural count was gathered from every 10th sections to provide a 10% sample selection per ovary. (15) The number of corpora lutea and different classes of follicles in each ovary was recorded on the computer screen using stereo investigator Motic software (Motic images plus 2.0). Ovarian follicle classes were based on the classification of Junqueira et al., (16). Briefly, follicles were classified as "primordial" if they contained an oocyte surrounded by a partial or complete layer of squamous granulosa cells. "Primary" follicles showed a single layer of cuboidal granulose cells. "Growing" follicles had an oocyte surrounded by a multilayered, solid mantle of granulosa cells. "Secondary" follicles were surrounded by more than one layer of cuboidal granulosa cells and accumulations of follicular fluid appear between the granulosa cells. "Antral" follicles were characterized by a central oocyte enriched by a fluid-filled space and bordered by hundreds of layered granulosa cells.

Estimation of the number of follicles in volume unit of ovary (Nv) was calculated according to the following equation: Nv = ΣQ/[(a/f)×h]

in which "ΣQ"=number of follicles counted in 10 sections from each specimen, "h" is distance between selected sections and a/f was calculated from area/field .

### Electron microscopy preparation

For transmission electron microscopy, the right ovarian samples obtained from mice in each group were cut into 1 mm^3^ and fixed in 2% glutaraldehyde in a 0.1M phosphate buffer (Proscitech, Thuringowa, Australia) and postfixed in 1% aqueous osmium tetroxide (TAAB, Berkshire, UK). The pieces were then dehydrated through graded concentration of ethanol, and embedded in resin. One micron semi-thin sections were stained with toluidine blue. Ultra-thin sections from selected blocks were stained with uranyl acetate and lead citrate and observed in a LEO 906 type transmission electron microscope (Oberkochen, Germany).

### Statistical analysis

Data were subjected to analysis by SPSS 17.0 software (SPSS Inc., Chicago, IL, USA). All data were tested for homogeneity of variances by Levene static test. When the variances were homogenous, the number of different follicles as well as corpora lutea between the control and copper-treated mice on days 14 and 35 were separately analyzed by independent sample t-test. (17) Results were expressed as mean±SEM. Values were considered to be statistically significant at P<0.05.

## Results

### Serum Cu evaluation

Mean±SEM of serum Cu concentration was 14±0.6 µmol/L in the control group. There was not any significant difference between the serum Cu level after 14 (mean±SEM, 11±1.2 µmol/L) and 35 (mean±SEM, 19±2 µmol/L) days of copper sulfate administration (100 mg/kg) and the control group. Also, we could not find significant difference 14 days following administration of copper sulfate (mean±SEM, 22±2.3 µmol/L) at a dose of 200 mg/kg with the control group. Hence, significant difference was seen after 35 days (mean±SEM, 82±9.9 µmol/L).

### Morphometery of ovarian structures

The mean number of different types of follicles and corpus luteum in each experimental group on days 14 and 35 after copper sulfate administration are presented in Table 1. It was observed that only the number of antral follicles was decreased by 100 mg/kg copper sulfate administration following 14 days compared to the control group (7.24±0.91 vs. 13.23±2.41 respectively, P=0.043). Hence, mice ovaries treated with copper sulfate at a dose of 200 mg/kg after 35 days showed significantly lower quantities (mean±SEM) of all follicular classes including primordial, primary, growing, secondary, antral and also corpus luteum than the control group (2.18±0.11, 1.89±0.47, 3.41±0.19, 1.22±0.18 and 2.00±0.31 vs. 27.79±5.22, 22.49±2.51, 26.46±0.83, 19.85±3.77, 13.23±2.41 and 21.61±7.04 respectively). The histomorphometrical study revealed that treatment of mice with copper sulfate at a dose of 200 mg/kg was significantly able to reduce the mean number of all classes of follicles and corpus luteum even on day 14 and/or 35.

**Table 1:** Mean±SEM number of different classes of follicles and corpus luteum in the control and treated groups on days 14 and 35 following copper administration.

The days after copper administration	Groups	No. mice	Primordial follicles	Primary follicles	Growing follicles	Secondary follicles	Antral follicles	Corpus luteum
**14^th^ day**	Control	6	27.79 (5.22)	22.49 (2.51)	26.46 (0.83)	19.85 (3.77)	13.23 (2.41)	21.61 (7.04)
	Cu100	6	30.88 (8.80)	26.09 (4.58)	28.99 (7.27)	21.25 (4.80)	7.24 (0.91)*	24.64 (4.70)
	Cu200	6	2.18 (0.11)***	1.89 (0.47)***	3.41 (0.19)***	1.45 (0.69)***	1.22 (0.18)***	2.00 (0.31)***
**35^th^ day**	Cu100	6	3.90 (0.31)***	4.53 (0.71)***	4.45 (0.46)***	2.08 (0.23)***	1.69 (0.08)***	2.55 (0.15)**
	Cu200	6	2.89 (0.41)***	2.71 (0.68)***	3.01 (0.20)***	2.40 (0.51)***	1.42 (0.37)***	2.40 (0.42)**

Cu, Copper; Cu100, 100 mg/kg copper sulphate administration; Cu200, 200 mg/kg copper sulphate administration.

^*^Shows significant difference with the control group at a level of P<0.05.

^**^Shows significant difference with the control group at a level of P<0.01.

^***^Shows significant difference with the control group at a level of P<0.001.

### Electron microscopy 

**Fig. 1:**
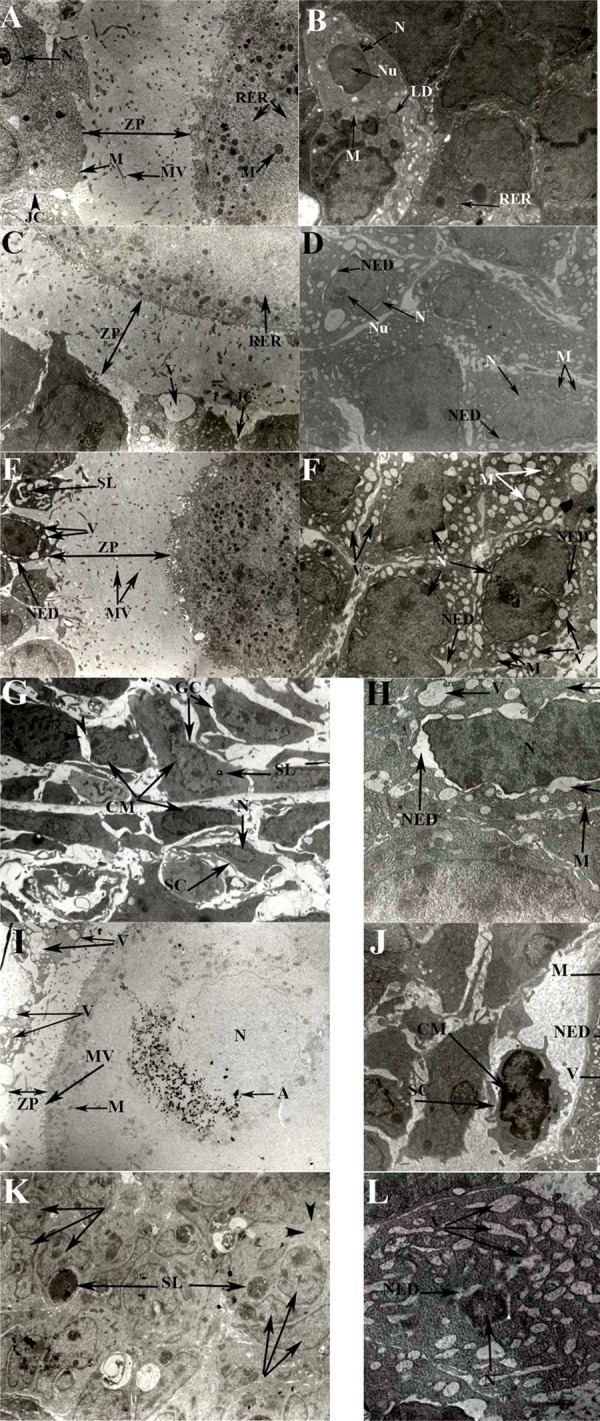
Electronmicrographs of the ovarian cells in the control (A & B) and treated animals including: 100 mg/kg copper sulfate (Cu100) for 14 days (C & D), 200 mg/kg copper sulfate (Cu200) for 14 days (E & F), 100 mg/kg copper sulfate for 35 days (G & H) and 200 mg/kg copper sulfate for 35 days (I, J, K & L). A: Healthy intracellular organelles in a growing follicle. Nuclei (N) of corona radiate cell and distinct and intact intercellular junction complex (JC) between them as well as normal mitochondria (M). Thickness of zona pellucida (ZP) and its microvilli distribution (MV) is normal as well as mitochondria and rough endoplasmic reticulum (RER) inside of oocyte (magnification=× 3000). Granulosa cell nuclei (N) with euchromatin appearance, prominent nucleolus (Nu) and normal organelles including RER, M and lipid droplet (LD), (magnification=× 3000). C: Damaged growing follicle from Cu100 experimental group after 14 days. Thickness of ZP and distribution of microvillus resemble lower than the control group. Vacuolated (V) area was seen within the cytoplasm of corona radiate. JC is still observable between corona radiate cells (magnification=× 3500). D: Luteal cells from Cu100 experimental group following 14 days. Mitochondria are often normal but nuclear enveloped dilations (NED) is obvious (magnification=× 5000). E: Growing follicle from Cu200 experimental group on day 14. Microvillus reduction and damaged JC are observable. Secondary lysosome (SL), vacuolated (V) organelles and NED of corona radiata cells show some sign of damage to these cells (magnification=× 3500). F: Luteal cells from Cu200 experimental group on day 14 show irregular nucleus with marginated chromatin and NED as well as several vacuolated (V) areas and damaged mitochondria (M), (magnification=× 5000). G: Irregular nucleus in granulosa cells (GC) and stromal cells (SC) of ovary with marginated chromatin (CM) and secondary lysosome (SL) on day 35 from Cu100 experimental group. Loss of intercellular junction complex (arrow heads) is seen (magnification=× 3000). Nuclei of corpus luteum with apparent chromatin segmentation, margination and condensation as well as abnormal mitochondria, extended NED and several vacuolated (V) areas on day 35 from Cu100 experimental group (magnification=× 7500). I: An oocyte on day 35 in Cu200 experimental group. Thickness of ZP resembles thinner and microvilli (MV) are lower than the control group. Corona radiata cell layer has been completely damaged. Several vacuolated (V) areas within cytoplasm and loss of intercellular junction complex are obvious. A, artifact (magnification=× 3000). J: On day 35 in Cu200 experimental group, heterochromatin nucleus with chromatin margination (CM) and several vacuolated (V) areas within the cytoplasm of granulosa and stromal cell (SC) is apparent. Nuclear enveloped dilation (NED) has been induced and mitochondria has been enlarged and damaged (magnification=× 3000). K: Corpus luteum on day 35 after receiving 200 mg/kg cooper sulfate. Note the decrease in organelles within cytoplasm (arrow heads), irregular and segmented nucleus (arrow). Secondary lysosome (SL) containing several apoptotic bodies (magnification=× 5500) & L: Corpus luteum on day 35 after receiving 200 mg/kg copper sulfate. Nucleus (N) with condensed chromatin and several vacuolated (V) areas within cytoplasm. Intercellular matrix has been lost (arrow heads). Nucleus enveloped dilation (NED) is apparent (magnification=× 8500).

In the ovaries of the control group, thickness of zona pellucida and distribution of microvillus as well as the other organelles like mitochondria and rough endoplasmic reticulum was normal. Intercellular junction complex between corona radiata cells was intact. The nuclei of granulosa cells expressed euchromatin appearance and prominent nucleolus. Normal mitochondria, rough endoplasmic reticulum and lipid droplet was obviously seen within the cytoplasm of granulosa cells (Fig. 1A & B). 

Following 14 days of 100 mg/kg Cu receiving (Cu100 experimental group), thickness of zona pellucida and distribution of microvilli was negatively affected and vacuolated area was seen but still intercellular junction complex was observable. Nuclei of luteal cells was euchromatin with obvious nucleolus and still mitochondria were normal. However, nuclear envelop dilation was the common finding of Cu damages which was seen after 14 days even with low dose of copper (100 mg/kg, Fig. 1C & D). 

Administration of 200 mg/kg copper sulfate for 14 days induced more damages to cells including the presence of secondary lysosome, some vacuolated organelles and nuclear envelop dilation within the cytoplasm of corona radiata cells as well as irregular nucleus with marginated chromatin and several vacuolated area within cytoplasm of luteal cells (Fig. 1E & F). 

The signs of cell damages with 100mg/kg copper sulfate consumption on day 35 were similar to 200mg/kg Cu for 14 days (Fig. G & H).

Hence, severe cellular changes were observed in Cu 200 experimental group on day 35 (Fig. 1I,J,K & L).

Corona radiata cells layer was completely damaged. Zona pellucida thickening resembled thinner than the control group. Decline in the number of cytoplasmic organelles as well as losing of intercellular matrix and extended intracellular vacuolaion were another changes. More secondary lysosomes within the cytoplasm containing much apoptic bodies were another finding. The other interesting observation was irregular cell shape and segmented nuclei. Nuclei had condensed chromatin and chromatin margination was apparent. Thus, more enlarged and damaged mitochondria were seen in the cytoplasm.

## Discussion

The results of the present study clearly demonstrated that short term administration of Cu (14 days) even with low dose (100 mg/kg) is able to have deleterious effects on intracellular organelles of rat ovarian cells. Interestingly, at this time serum Cu level or even the number of corpora lutea and follicles did not have any significant changes. However sever ultrastructural damages was seen following 35 days at a dose of 200 mg/kg which was coincident with significant increase in serum Cu level. Although according to the morphometrical studies, beforehand all kind of follicles had been significantly decreased in number. These findings are in agreement with the previous report, (18) who demonstrated that Cu was both genotoxic and mutagenic when mice were gavaged for 6 consecutive days (subchronic exposure) with 8.5 mg/kg copper containing water.

The metabolism of Cu involves mainly its transfer to and from various ligands, most notably sulfhydryl and imidazole groups on amino acids and proteins. The liver is the most important organ involved in Cu disposition. It serves as a storage depot of Cu which is then released into the blood or as a site for the formation of various Cu complexes for subsequent biliary excretion. Biliary Cu is returned to the intestine and excreted in the feces. (19) In chronic copper poisoning, Cu is gradually deposited in the liver without producing any significant clinical sign and/or serum Cu change, so makes an early diagnosis very difficult. (1) The similar finding was observed in our study in which elevation in serum Cu was not seen at the first 14 days and instead of antral follicles, the other kind of follicles did not show any significant changes. Therefore, the results of this study suggest that antral follicles might be more susceptible to copper overexposure and undergo atresia or produce more corpora lutea as a result of ovulation. Chronic exposure of rats to copper chloride fumes show decreased concentrations of plasma FSH, LH and testosterone and dysfunction of gonads. (20) It is well known that ovarian follicles following antrum formation are gonadotrophin dependent and their development and atresia are influenced by FSH, (21) and LH. (22) However, Murawski et al., (12) reported that continuous exposure to copper had no effect on the growth parameters of follicles. On the other hand, Early studies showed that Cu administration induced ovulation in rabbits. (10) According to our results we believe that Cu can affect follicles at the beginning of consumption probably as a result of hormonal variations. Because, elevated levels of Cu are associated with increased epinephrine and dopamine synthesis in brain and release of neurotransmitters is also influenced by this metallic ion, (23) and lead to induction of atresia. (24)

The other possible explanation for follicular damage can be due to effects of Cu on cell apoptosis. Apoptosis is associated with specific morphological changes which are characterized by chromatin condensation, nuclear DNA fragmentation, cell shrinkage and membrane-enclosed cell fragment (apoptic body) formation. (25,26) Some of these changes such as chromatin condensation and margination, nuclear segmentation or formation of apoptosis body were observed in our experiment specially following long term Cu administration. Several researchers have reported Cu cytotoxicity in a variety of in vitro and in vivo systems. Wataha, (27) observed that the reduction of cell proliferation and increased frequency of nonviable cells are dependent on copper concentration. It has also been shown that copper compounds delay cell-cycle progression and increase cell death in different cell cultures. (28-30) Previous investigations provide evidence that Cu ions are capable of interacting directly with nuclear proteins and DNA causing site-specific damage. (31) Copper easily reacts with both individual amino acids and proteins containing histidine and cysteine. Complexes of Cu ion with amino acids or peptides are able to bind to DNA forming Cu-amino acid-DNA complexes which in consequence leads to constant change of the structure genes. Modulation of transcription is exerted by copper's influence on the properties of the transcription factors. (32) Copper binds to DNA with higher affinity than other cations and thus promotes DNA oxidation, (33) catalyses efficient formation of 8-aminodeoxyguanosine as well as 8-oxidG, (34) and causes apoptosis in cultured cells. (35,36)

There are the other possible explanation for inducing cell damage by Cu. Copper is a strong oxidant, in which it could bind to cell molecules during the high load. (37) Indirectly they can catalyse through Fenton/Haber-Weiss chemistry the production of cell damage as a consequence of metal-driven formation of reactive oxygen species (ROS). (38,39) For example, it has been reported that dietary copper overload in rats has been caused the lipid peroxidation of mitochondrial membranes. (40) In addition, ions from metals such as Cu exhibit high affinity for thiol groups and may therefore severely disturb many cell metabolic function. (41) Consequently, oxidative stress that is the state of redox disequilibrium in which ROS production overwhelms the antioxidant defense capacity of the cell, may lead to adverse biological consequences such as damage to lipids, DNA or proteins resulting in excess cell proliferation, apoptosis, or mutagenesis. (42)

One of the most observed phenomenon in the present ultrastructural study of cells treated with Cu was formation of vacuole within the cytoplasm organelles. Vacuolization of cytoplasm organelles and detachment of cell membrane from its basement membrane as were seen in our study are some evidence of cell destruction process. (43) It is well shown that neurodegenerative diseases increase apoptosis rate and reduce ATP synthesis and are associated with mitochondrial vacuolization and dissolution of their cristae. (44)

In conclusion, the present work provides new evidence of ultrastructural features of ovarian cells as well as morphometrical study of different follicles and corpus luteum following short and long term copper exposure. Even though, the adverse effects of copper on intracellular organelles suggested early ovarian cell damage but according to the morphometrical results, only antral follicles was the most susceptible cells that showed significant reduction with the lower and shorter copper consumption. Nevertheless, long term or higher dose of copper affected the whole of ovarian structures morphmetrically.
